# A Case of Atorvastatin-Associated Necrotizing Autoimmune Myopathy, Mimicking Idiopathic Polymyositis

**DOI:** 10.1155/2018/5931046

**Published:** 2018-06-20

**Authors:** Ayushi Dixit, Adriana Abrudescu

**Affiliations:** Icahn School of Medicine at Mount Sinai-Queens Hospital Center, Queens, NY, USA

## Abstract

Statin-induced necrotizing autoimmune myopathy (SINAM) is a rare side effect of statin use which manifests as progressive muscle weakness. Because statins are a widely prescribed medication for coronary artery disease, hyperlipidemia, and many other diseases, many patients are at risk of developing SINAM or one of the many other statin-induced myopathies. Due to identification of an antibody specific to this disease, we were able to diagnose SINAM in a patient whose symptoms had progressed to the extent that they were debilitating. Our case describes SINAM in a patient undergoing treatment with a statin for an extended period of time, diagnosis of the disease process, treatment, and resolution of symptoms.

## 1. Introduction

Statins are 3-hydroxy-3-methylglutaryl-coenzyme A reductase inhibitors and are some of the most widely prescribed medications for treating atherosclerotic disease in order to reduce the morbidity and mortality for both coronary and cerebral vascular diseases. Most statin-induced myotoxicity is self-limiting with myalgia and transient elevation of muscle enzymes followed by complete recovery within weeks to months upon the discontinuation of the statin. Statin-induced necrotizing autoimmune myopathy (SINAM) is a rare side effect reported in about 2-3 out of 100,000 people who use statins. We present a case of SINAM manifested in a patient with over ten years of simvastatin use who was recently switched to atorvastatin.

## 2. Case Presentation

**Figure 1:**
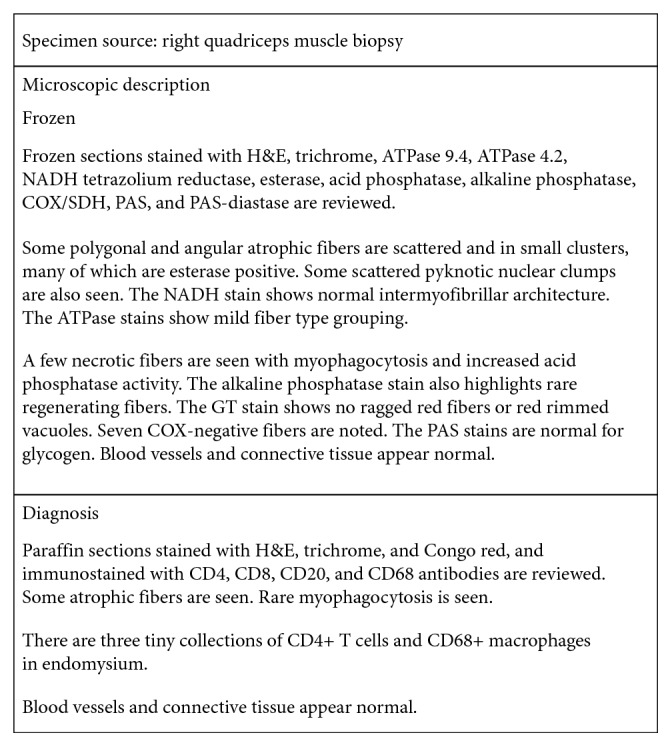
Surgical pathology report.

**Figure 2:**
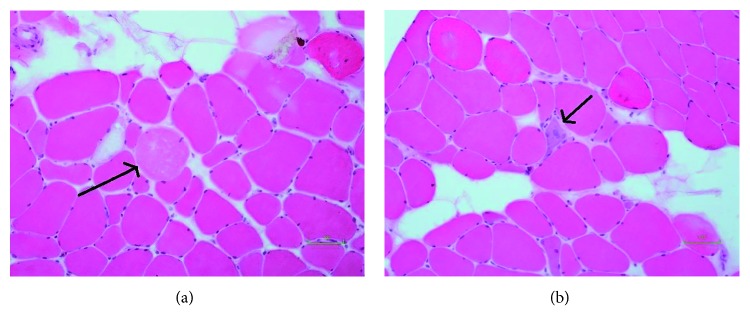
Microscopic right quadriceps muscle biopsy showing necrotic muscle fibers and regenerating fibers. (a) Arrow pointing at necrotic muscle fiber (magnification 20x with alkaline phosphatase stain). (b) Arrow pointing at regenerating muscle fiber (magnification 20x with alkaline phosphatase stain).

A 66-year-old female with a medical history of hypertension, hyperlipidemia, diabetes mellitus type 2, and obesity presented with hip pain after a fall. Radiograph of the hip was negative for dislocation or fracture. On physical exam, she had proximal muscle weakness greater in the lower extremities, and the rest of her examination was negative for skin rashes, lymphadenopathy, joints inflammation, and pedal edema. Her EKG showed normal sinus rhythm with no ST-T changes. Blood work showed elevated CPK (9767), CKMB (101.50), and aldolase (60) levels and LFT derangement (AST-302 and ALT404) (Tables 1 and 2). Other lab findings were negative for ANA, anti-Jo antibody, and antimitochondrial antibody. Thyroid function tests were normal. At that time the patient was taking atorvastatin 80 mg which recently replaced simvastatin for better hyperlipidemia control. Atorvastatin was discontinued, and the patient was followed closely in the medical clinic. The proximal muscle weakness continued to progress rapidly during the following month with significant impairment of her walking and rising from a chair without support. The physical exam revealed a decrease to 2 out of 5 motor strength of the proximal upper extremities and 3 out 5 of the proximal lower extremities, and the rest of the physical examination remained normal. Laboratory results with persisting elevated muscle enzyme (CPK, CKMB, and aldolase) levels remained elevated. Testing for anti-HMGCR antibody with ELISA was positive at 34 (reference value: greater than 20 is positive). Muscle biopsy was consistent with necrotizing myopathy (Figures 1 and 2). Taken with the antibody results, the final diagnosis of statin-induced necrotising autoimmune myopathy was performed. Findings were consistent with diagnosis of necrotizing autoimmune myositis. Prednisone therapy started at 1 mg/kg per day and tapered over the next three months. The patient's proximal upper and lower strength and mobility improved back to her baseline with normalization of the muscle enzymes and the liver function tests. Upon further review of the patient's chart it was found that she had been taking simvastatin for at least thirteen years which was recently discontinued after which she was switched to atorvastatin. Due to necrotizing myopathy's high association with malignancy, cancer screening reports were reviewed. The patient had a normal colonoscopy in 2014, cervical cancer screening was negative, and calcifications on serial mammograms remained stable. The patient started complaining of weakness one month after initiation of the new therapy with atorvastatin. Liver function tests recorded during treatment with simvastatin were within normal limits.

## 3. Discussion

Since their introduction over 20 years ago, statins (3-hydroxy-3-methylglutaryl-coenzyme A reductase inhibitors) have been one of the most widely prescribed medications, and treatment with statins has effectively reduced the cardiovascular morbidity and mortality. Evidence presented by the American College of Cardiology/American Heart Association Blood Cholesterol Treatment Task Force solidified statins as the only cholesterol-lowering agent that showed mortality benefit. The new guidelines have expanded the statin-eligible population in the United States from 43 to 56 million, a total increase of 13 million. This number further approaches 1 billion worldwide [1]. Muscular side effects of statins are diverse, ranging from common mild myalgia with localized weakness to life-threatening rhabdomyolysis [2]. Myopathic symptoms produced by statins generally resolve within weeks to several months of stopping the medication [3]. Recently a new entity called statin-induced necrotizing autoimmune myopathy (SINAM) was identified and has led to identification of an autoantibody against 3-hydroxy-3-methylglutaryl coenzyme A reductase [4]. The novel anti-HMGCR antibody, which was discovered in 2010, is a promising diagnostic marker for statin-associated NAM. Almost all patients with statin-induced necrotizing autoimmune myopathy have tested positive for anti-HMGCR antibodies. The sensitivity and specificity of the anti-HMGCR antibodies are 94.4% and 99.3%, respectively [5, 6]. This new entity was recently described by Mammen et al. who first identified a group of 16 patients with necrotizing myopathy associated with a new antibody recognizing 200 kD and 100 kD proteins [5]. SINAM is an autoimmune disease that requires specific therapy and possibly long-term immunosuppression. Different statins have varied propensities for causing self-limited myopathy, with atorvastatin and simvastatin associated with higher rates of myopathy than rosuvastatin; however, there is no established association between specific statins and the occurrence of SINAM [7, 8]. A study by Troyanov involving 12 patients with exposure to atorvastatin and diagnosed with atorvastatin-induced autoimmune myopathy reported that the mean interval between atorvastatin discontinuation and diagnosis of the myopathy was 17.8 months (range of 0–79 months) [9]. His study was performed at a tertiary care center, and response to steroid therapy was inadequate subsequently resulting in therapy with methotrexate and azathioprine. Our patient's dramatic response and improvement may be attributed to the fact that SINAM was identified two months after initiation of atorvastatin. Our case is the second one reporting a diagnosis of SINAM after changing simvastatin to atorvastatin, the first being reported by Nichols et al. [5]. As of yet, there are no control trials to guide treatment. In the past, treatments with high doses of steroids, methotrexate, and azathioprine have been given with variable responses. Our patient responded to a three-month course of steroids. We believe our case is unique because the early recognition of HMGCR antibodies and prompt treatment resulted in full recovery.

## 4. Conclusion

As statins are an extensively used class of drugs, clinicians should be aware of SINAM and patients should be promptly evaluated at the earliest signs of inflammatory muscle disease which mimics polymyositis. It is important to differentiate statin-associated inflammatory myopathies from other self-limited myopathies, because the disease will not subside following discontinuation of the statin [10]. Muscle biopsy is crucial for differentiation; however, in the future, larger studies could prove whether using the HMGCR antibodies alone would be sufficient to make the diagnosis and implement the immunosuppressive or intravenous immunoglobulin therapy. Our case report illustrates the importance of early recognition of SINAM and that the treatment requires immunosuppressive agents to prevent permanent damage and it can result in complete recovery. Upon our review of literature, atorvastatin use was reported in cases of SINAM; however, there is still speculation about whether any one statin is linked with the disease. Further studies should be done to determine whether the use of atorvastatin, when compared with other statins, is more likely to result in a positive HMGCR antibody test.

## Figures and Tables

**Table 1:** Muscle enzyme trend from diagnosis to resolution of disease process.

	Reference range and units	3/24/2017	3/29/2017	6/20/2017	8/8/2017
Aldolase	Normal: 3.3–10.3 U/L	60.0	31.2	8.4	7.7
CK	Normal: 20–170 U/L	9767	6114	653	88
CKMB	Normal: 0.00–3.77 ng/ml	101.50	73.33	9.00	

**Table 2:** Liver function enzyme trend from diagnosis to resolution of disease process.

	Reference range and units	3/11/2017	3/12/2017	3/13/2017	3/15/2017	3/16/2017	4/25/2017	8/8/2017
Alkaline phosphatase	Normal: 40–120 U/L	105	110	117	109	102	71	79
ALT	Normal: 10–45 U/L	404	347	356	363	331	349	23
AST	Normal: 10–40 U/L	302	274	322	323	273	120	17
